# Diagnostic methods in ocular argyrosis: case report

**DOI:** 10.1007/s10633-016-9552-z

**Published:** 2016-07-08

**Authors:** Monika Sarnat-Kucharczyk, Dorota Pojda-Wilczek, Ewa Mrukwa-Kominek

**Affiliations:** Department of Ophthalmology, School of Medicine in Katowice, Medical University of Silesia, ul. Ceglana 35, 40-514 Katowice, Poland

**Keywords:** Silver nitrate, Argyria, Argyrosis, Ocular argyrosis

## Abstract

**Purpose:**

The aim of this report is to present a case of a patient, metal foundry worker, who had been exposed to industrial silver salts for over 20 years. It is well established that chronic exposure to silver compounds can cause accumulation of silver deposits in various tissues. This condition is referred to as argyrosis or argyria, whereas changes related to eye tissues are defined as ocular argyrosis.

**Methods:**

A complete eye examination, corneal confocal microscopy, kinetic and static visual field test, posterior segment optical coherent tomography, pattern visual evoked potentials (PVEP), flash visual evoked potentials, multifocal electroretinogram, pattern electroretinogram (PERG), full-field electroretinography (FERG) and electrooculogram were all performed.

**Results:**

Eye examination revealed decreased visual acuity, corneal deposits and drusenoid changes within the macula. Although electrophysiology tests did not show changes in the function of retinal pigment epithelium, they revealed abnormal function of photoreceptors in the central and peripheral retina. PERG abnormalities and delayed latency of P100 wave in PVEP confirmed impaired function of the inner layers of the retina in the macular region.

**Conclusions:**

Corneal confocal microscopy and electrophysiological tests may help confirm the diagnosis of ocular argyrosis.

## Introduction

Silver is a naturally occurring element with high thermal and electrical conductivity. It is harder than gold, very ductile and malleable, and it has antimicrobial effects.

The properties of silver have been known since antiquity. Currently, the metal is widely used in metallurgy, photography, medicine, and for the production of jewelry, coins and mirrors; it is also utilized as a water disinfectant [1].

Argyrosis involves the accumulation of silver in various tissues, leading to their dysfunction. Argyrosis is a rare dermatological and systemic disease caused by the accumulation of silver deposits mainly in the skin, mucous membranes and internal organs following chronic exposure to silver or silver compounds.

The disease can be diagnosed based on blood and urine tests. Correct diagnosis is facilitated by skin biopsy, which reveals small gray-brown or brown-black granules, approximately 1 mm in size, scattered mainly in the dermis and eccrine sweat glands [2].

Chronic exposure to silver compounds through ingestion, inhalation or skin contact results in the accumulation of silver in the eyes and in the adjacent tissues [3]. This condition is defined as ocular argyrosis, the most common type of localized argyria [4]. The disease may also develop following the use of silver-containing eye drops, certain eyelash and eyebrow dyes [5].

The deposition of silver salts results in skin discoloration around the eyes; the affected area becomes gray or bluish-gray. Silver precipitates can be distributed in the elastic fibers of the connective tissue and basement membranes [6] including the eyelids, conjunctiva, lacrimal sac, lens, ciliary body and Bruch’s membrane, but have also been noted within Bowman’s membrane, corneal stroma and Descemet’s membrane, causing its discoloration [7].

Ocular argyrosis diagnosed during a thorough ophthalmic examination may be the first sign of generalized argyria.

Adverse health outcomes of silver depend on the dose, duration and form of exposure (i.e., ingestion, inhalation or skin contact) as well as on individual characteristics (age, sex, nutritional status and general health).

Under normal circumstances, people are exposed to small amounts of silver present in food, drinking water, soil and sometimes also in the air.

Consumption of large doses of colloidal silver can cause coma, pleural edema and hemolysis. Silver is also toxic to bone marrow and may cause agranulocytosis. Ingestion of high doses of inorganic silver yields effects similar to those of corrosive solutions, i.e., burning of the throat and epigastric area leading to abdominal pain, vomiting and diarrhea. A single lethal dose of silver nitrate estimated by the World Health Organization is about 10 grams. Silver salts are more toxic than silver proteins or colloidal silver, very popular in alternative medicine [2].

## Case report

A 71-year-old patient was referred to the Corneal Unit of the University Eye Hospital in Katowice, Poland, due to gradual deterioration of distance and near visual acuity, night blindness (nyctalopia) being the most remarkable complaint in the last few years. He also had corneal changes in both eyes. The patient admitted having been exposed to industrial silver salts in a metal foundry where he had worked for over 20 years. He was retired for 5 years, and during that time, he was no longer exposed to silver.

Uncorrected distance visual acuity was 0.3 in the right eye (RE) and 0.4 in the left eye (LE) on the Snellen chart. Correction did not improve distance visual acuity. Best corrected near visual acuity was 1.0 for the RE with correction 2.5 Dsph and 0.75 for the LE with correction 2.5 Dsph. Intraocular pressure was 19 mm Hg and 17 mmHg in the RE and LE, respectively.

**Fig. 1:**
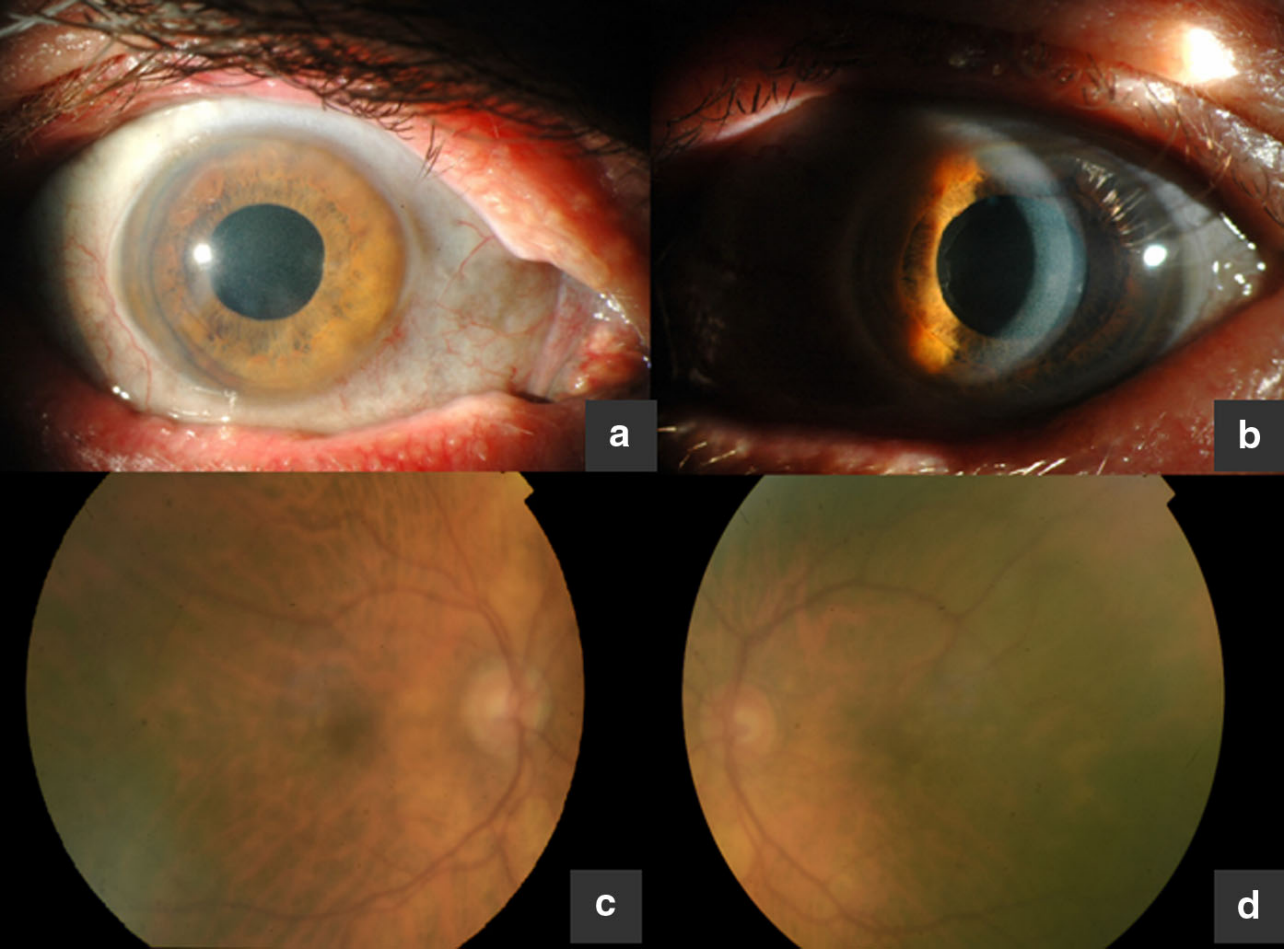
**a** Conjunctival discoloration, grayish-black pigmentation in the area of the medial canthus. **b** Silver deposits scattered in corneal layers, pseudoexfoliative syndrome. **c** Right eye fundus. **d** Left eye fundus

Slit lamp examination revealed discoloration of the eyelid skin and conjunctiva in the anterior segment, more prominent in the medial canthal region. Corneal stromal haze was present in the center and periphery, with confluent whitish gold-like deposits extending from the anterior and deep stroma to Descemet’s membrane. The patient also had nuclear cataract in the RE; his LE was pseudophakic with pseudoexfoliative syndrome. Dilated-pupil fundus examination showed drusenoid changes in the macular region and rearrangement in retinal pigment epithelium (RPE). Optic nerves were normal (Fig. 1). The skin of his neck and upper limbs showed bluish-gray discoloration.

## Results

### Corneal confocal microscopy

**Fig. 2:**
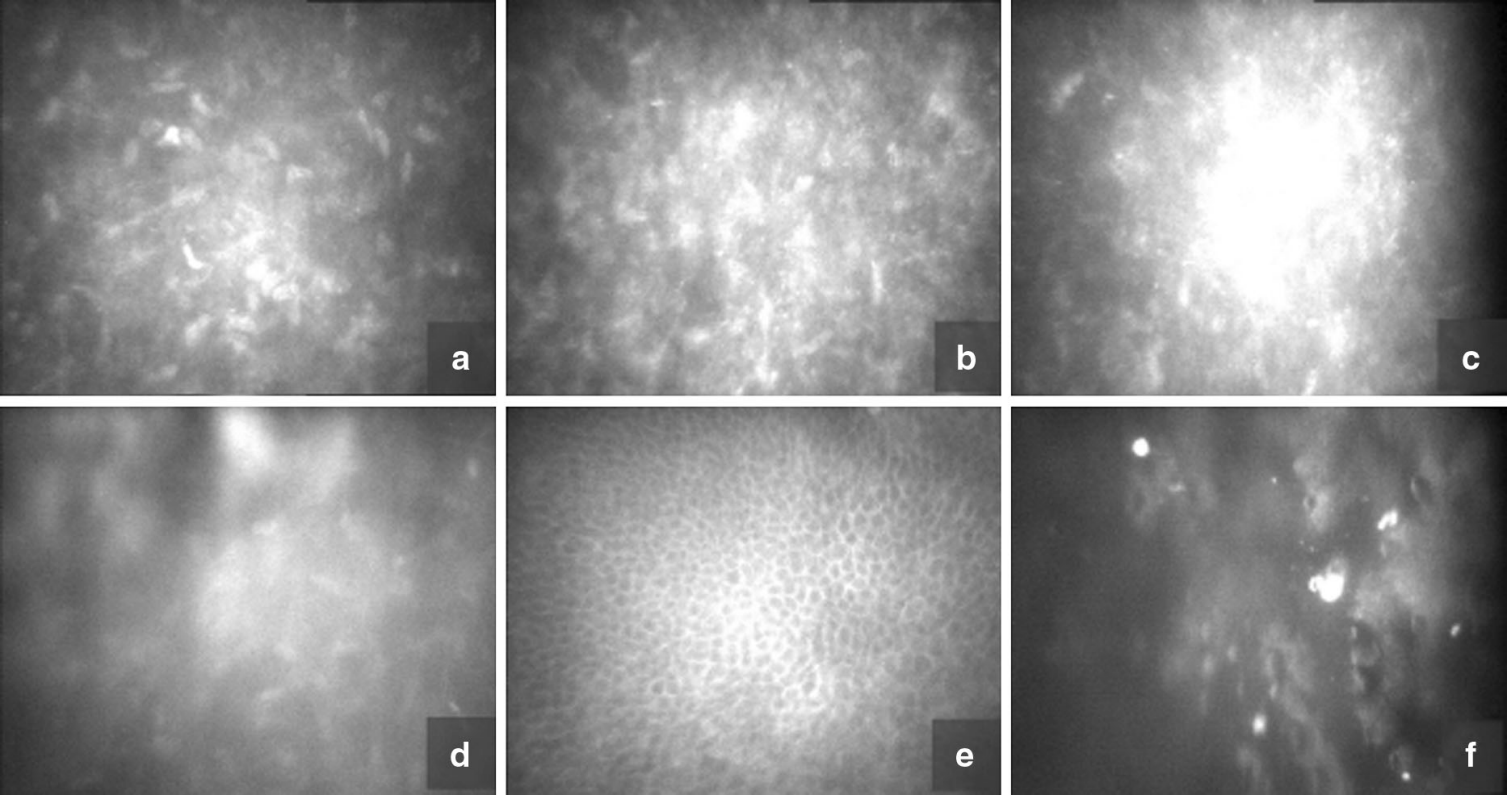
Corneal confocal microscopy. **a, b** Highly reflective punctiform deposits in the stroma, hyper-reflective keratocytes, changes in the density and shape of the keratocytes. **c, d** Confluent, dense deposits **e** Normal basal layer of corneal epithelium **f** Obscured view of corneal endothelium

Confocal microscopy, CS4 (Nidek Technologies, Italy), showed characteristic changes. Confluent small silver deposits were seen in the center and periphery of the cornea. Descemet’s membrane thickening was also found. Due to disease progression, numerous punctate hyper-reflective silver deposits hindered accurate endothelial cell assessment. Changes in the cornea resembled accumulated gold deposits (Fig. 2).

### Visual fields

**Fig. 3:**
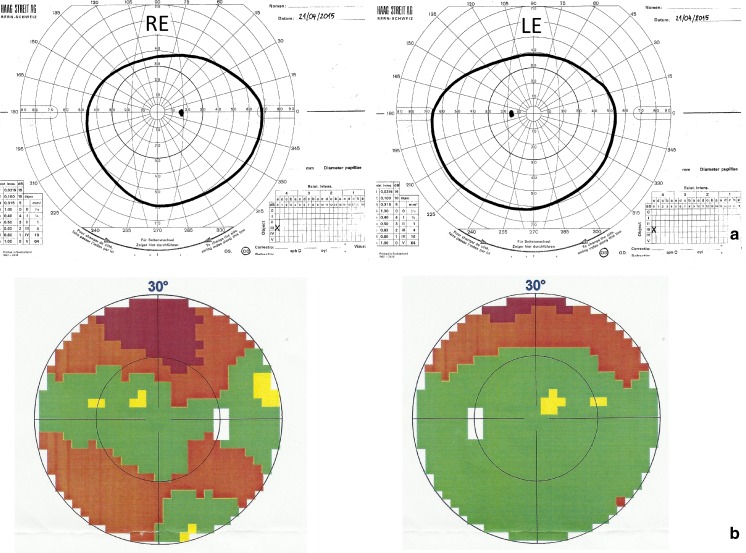
**a** Kinetic visual fields of the right and left eyes. **b** Static visual fields of the right and left eyes

Goldmann kinetic perimetry (Haag Streit, Switzerland) revealed visual field defects in the upper, nasal and temporal quadrants of the RE, while in the LE the loss was limited to upper and temporal quadrants. Octopus 1-2-3 static perimetry (Haag Streit, Switzerland) showed reduced retinal sensitivity and relative scotomata—predominantly in the RE (Fig. 3).

### Posterior segment optical coherent tomography (PS-OCT)

**Fig. 4:**
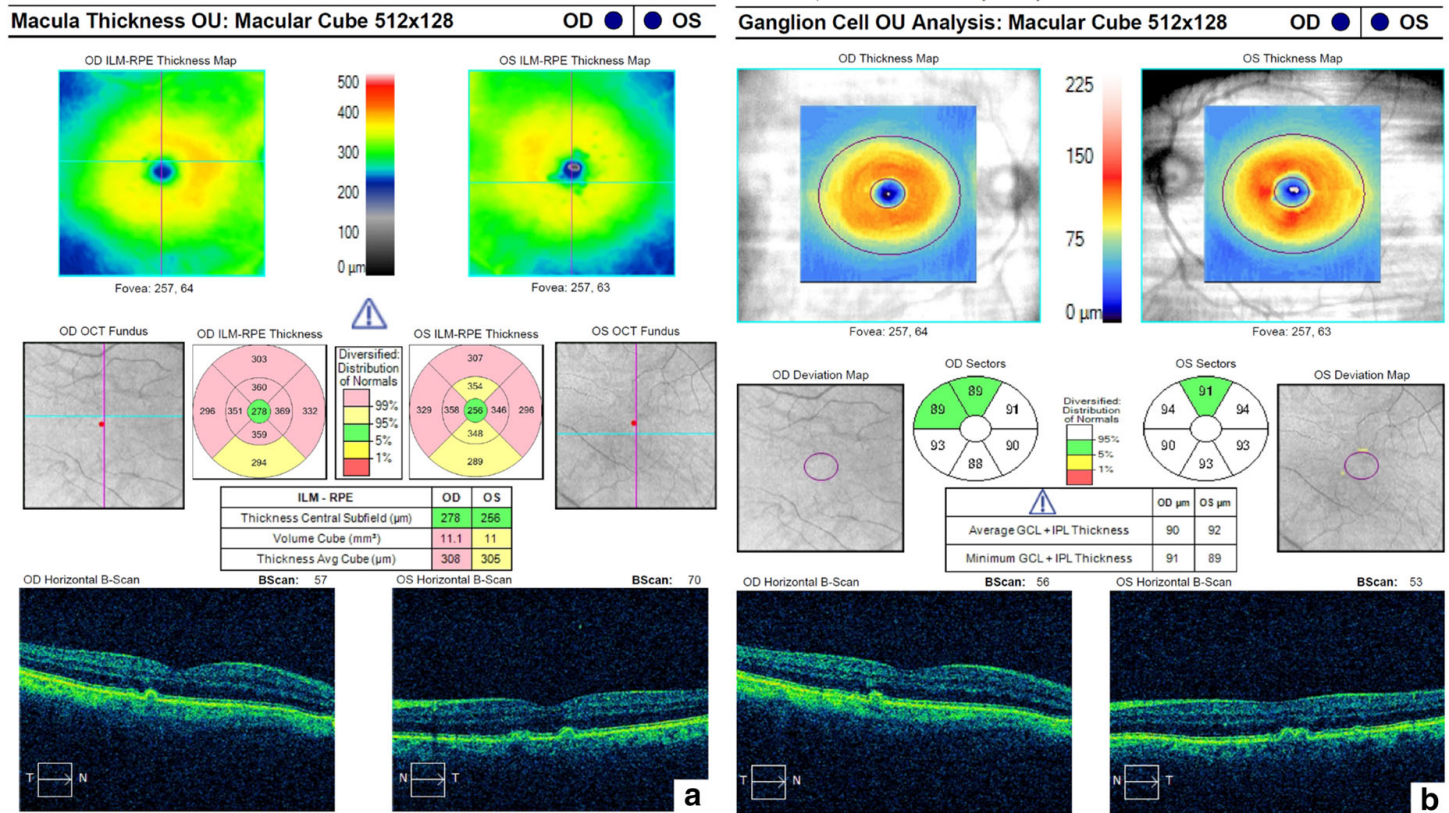
OCT of the right and left eyes. **a** Small retinal pigment detachment secondary to drusenoid changes in the macula. **b** Ganglion cells complex

PS-OCT (Cirrus HD-OCT, Carl Zeiss Meditec, Dublin, California) revealed drusenoid changes in RPE. The ganglion cells complex thickness map revealed a slight increase from the mean values (Fig. 4).

### Electrophysiological examinations (Reti-port and Reti-scan, Roland Consult, Germany; International Society for Vision and Eye Research standard protocols)

**Fig. 5:**
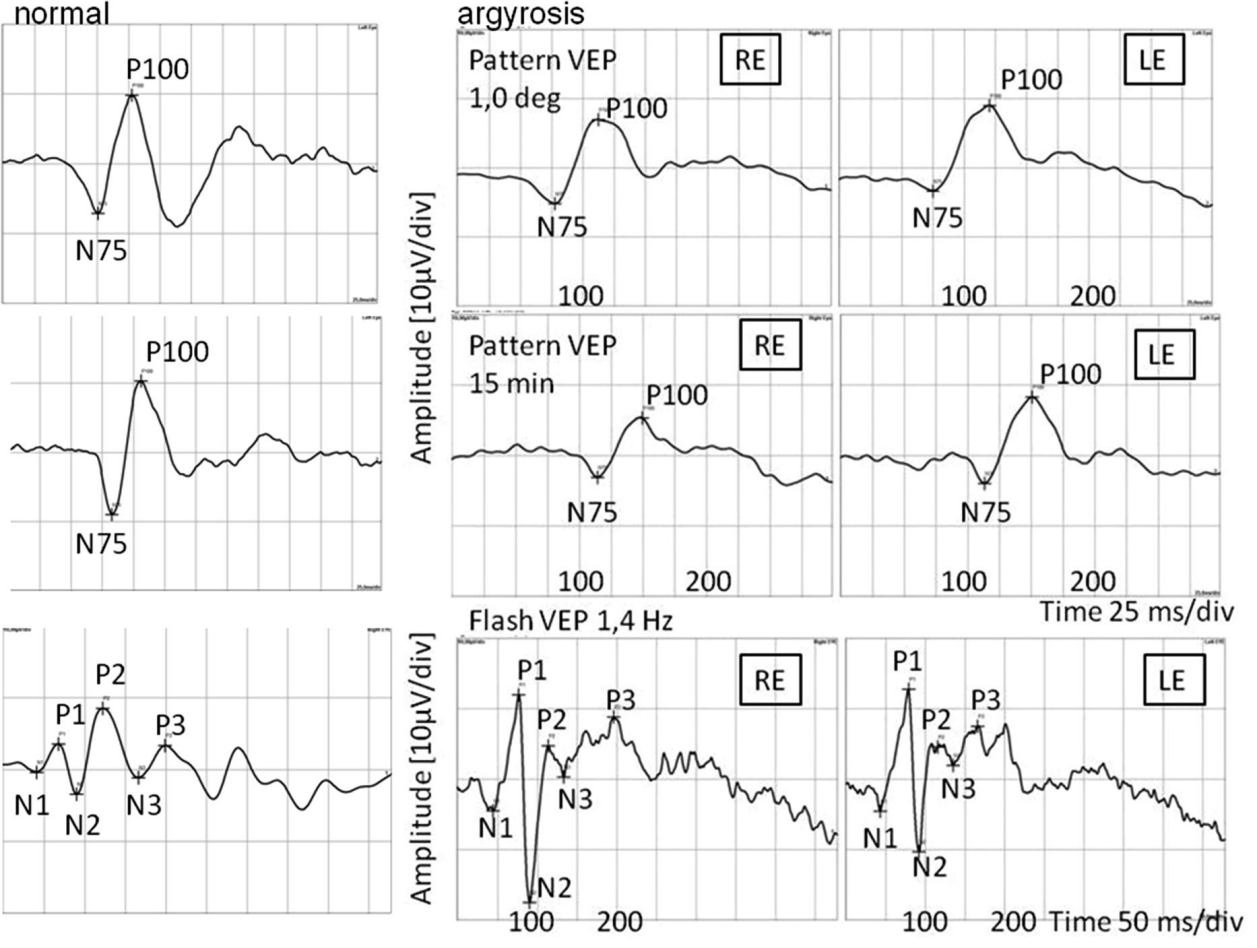
Pattern and flash VEP. *RE* right eye; *LE* left eye. Pattern VEP: P100 amplitude: 1.0° RE = 12.2 µV, LE = 12.4 µV (95 % reference interval: 7.0–9.2 µV); 15 min RE = 8.47 µV, LE = 12.3 µV (95 % reference interval: 6.2–9.4 µV); P100 latency: 1.0° RE = 113 ms, LE = 121 ms (95 % reference interval: 103.2–106.8 ms); 15 min RE = 150 ms, LE = 151 ms (95 % reference interval: 102–117 ms); flash VEP P2 latency RE = LE = 115 ms (95 % reference interval: 109.6–126.2 ms)

**Fig. 6:**
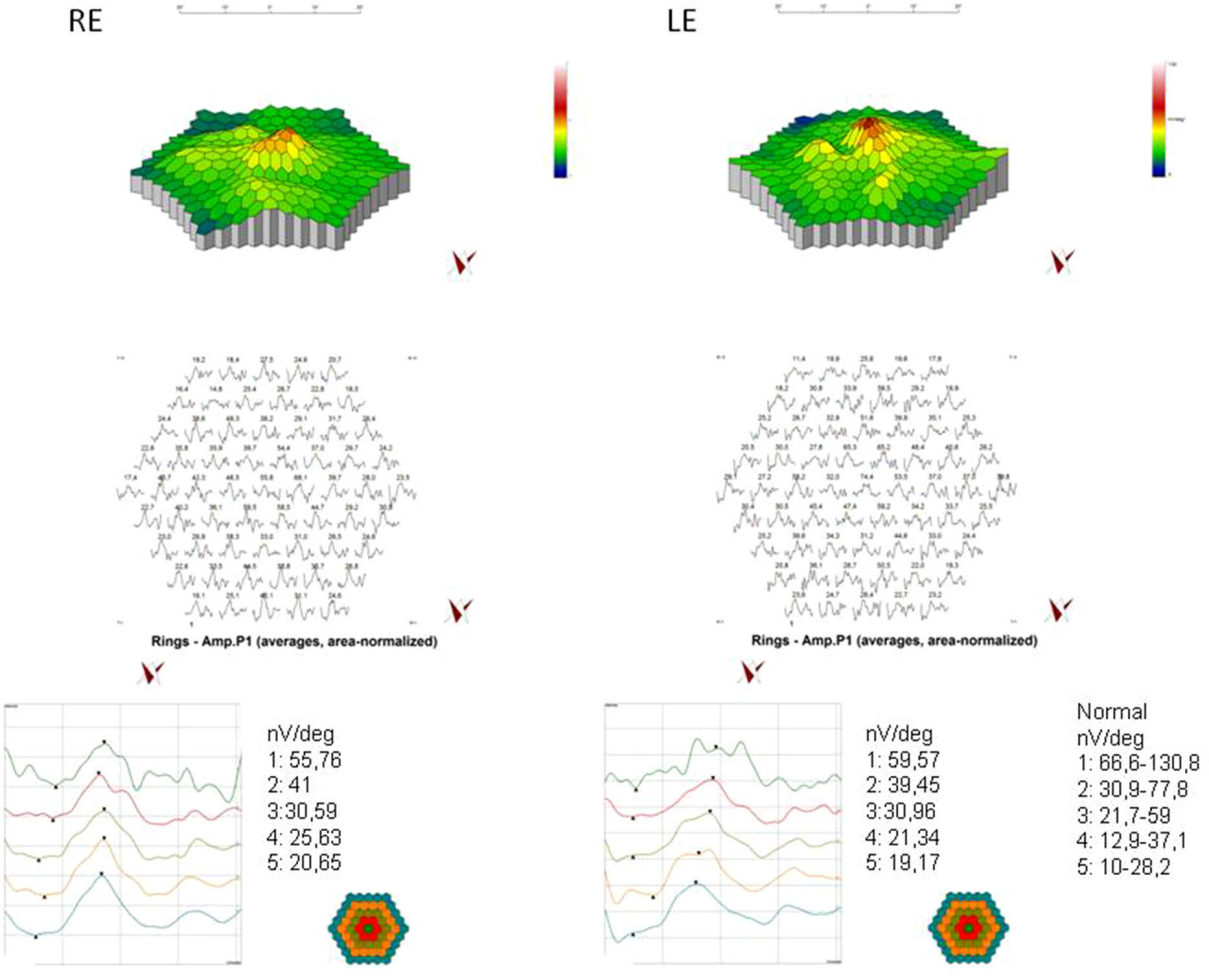
mfERG of the right and left eyes. *RE* right eye; *LE* left eye; P1 amplitude in rings

**Fig. 7:**
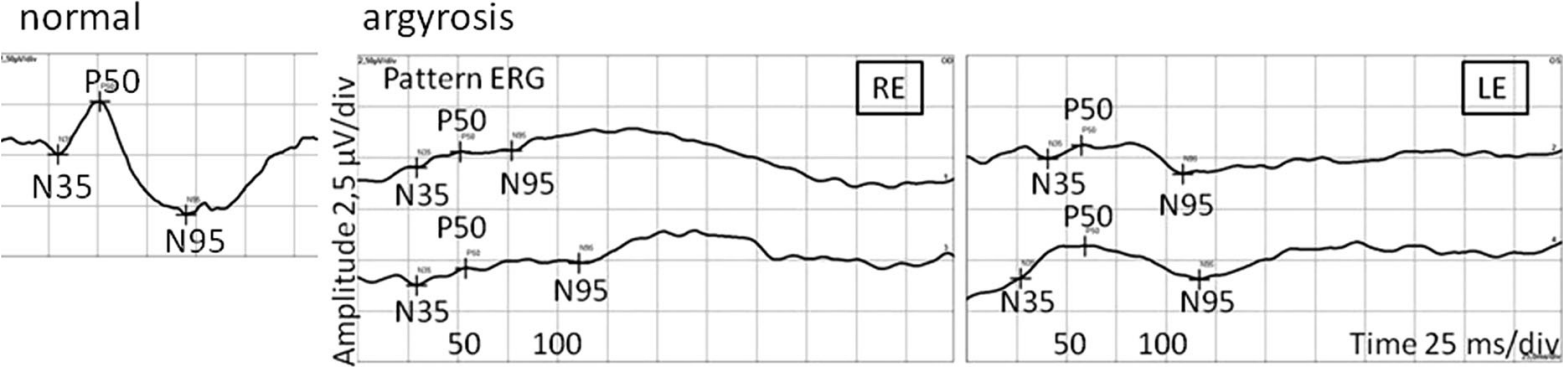
PERG of the right and left eyes. *RE* right eye; *LE* left eye; P50 amplitude: RE < 1 µV, LE = 1.58 µV (95 % reference interval: 1.4–2.6 µV); N95 amplitude RE < 1 µV, LE = 1.64 µV (95 % reference interval: 2.2–4.6 µV); P50 implicit time RE = 52 ms, LE = 59 ms (95 % reference interval: 49–54 ms); N95 implicit time RE = 111 ms; LE = 110 ms (95 % reference interval: 93–102 ms)

In pattern visual evoked potentials (PVEP), P100 latencies were delayed to 110 % after stimulation with 1°, and to 130 % after stimulation with 15′. P100 amplitudes were within normal limits. Flash visual evoked potentials (FVEP) revealed normal amplitudes and latencies (Fig. 5). Multifocal electroretinogram (mfERG) revealed reduced amplitudes of P1 wave in the first ring (Fig. 6). Pattern electroretinogram (PERG) was markedly abnormal with delayed N95 implicit time and reduced amplitudes of P50 and N95 waves (Fig. 7).

**Fig. 8:**
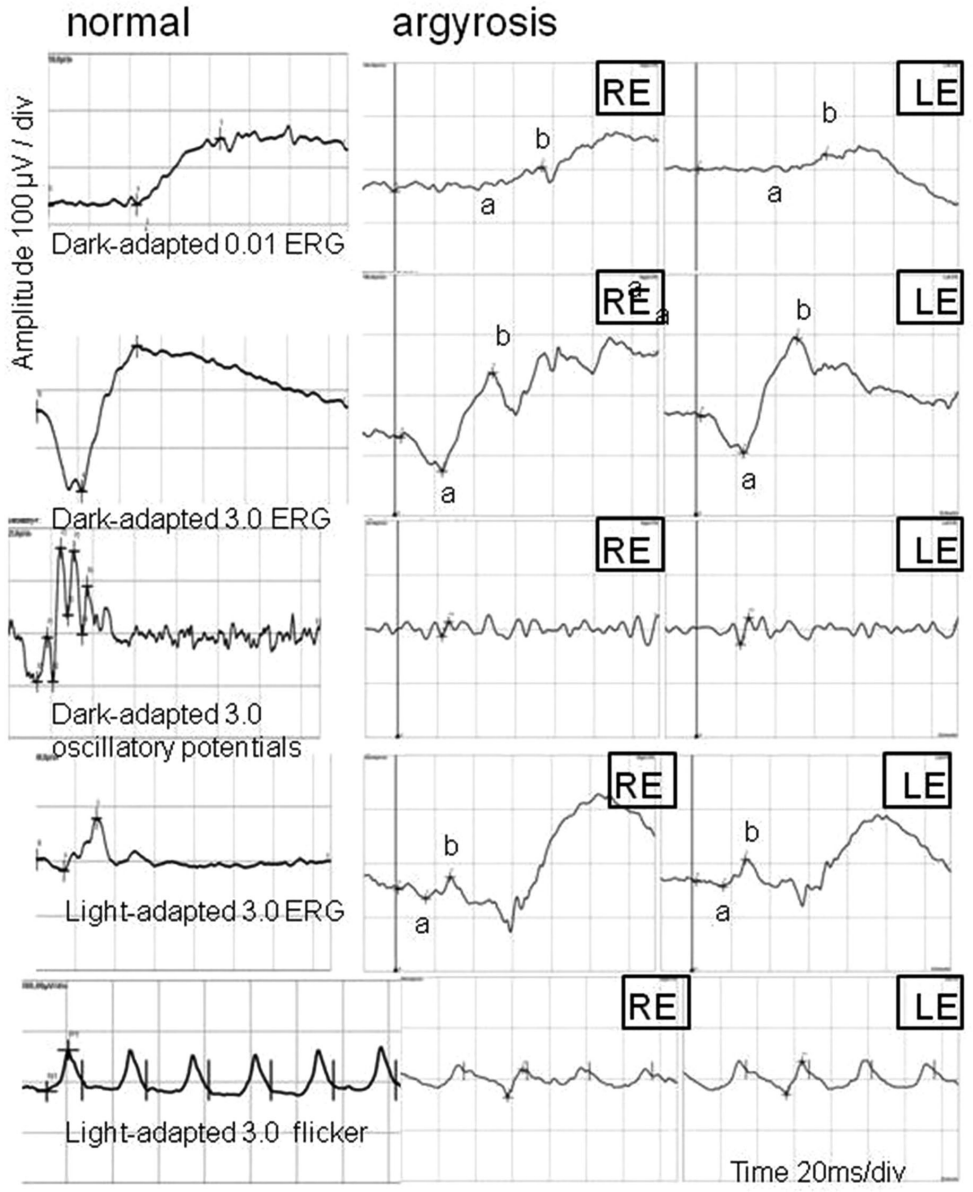
Full-field ERG of the right and left eyes. *RE* right eye; *LE* left eye; RE/LE: dark-adapted 0.01 ERG b-wave amplitude 46/27 µV (95 % reference interval: 95–305 µV); implicit time 75/67 ms (95 % reference interval: 67–91 ms); dark-adapted 3.0 ERG a-wave amplitude 56/60 µV (95 % reference interval: 155–356 µV); b-wave amplitude 163/190 µV (95 % reference interval: 290–654 µV); a-wave implicit time 24 ms (95 % reference interval: 14–22 ms); b-wave implicit time 50 ms (95 % reference interval: 33–46 ms); OP2 (oscillatory potential) amplitude 7/12 µV (95 % reference interval: 21–46 µV); OP2 implicit time 26 ms (95 % reference interval: 22–24 ms); light-adapted 3.0 ERG a-wave amplitude 17/9 µV (95 % reference interval: 26–62 µV); b-wave amplitude 40/50 µV (95 % reference interval: 103–250 µV); a-wave implicit time 16 ms (95 % reference interval: 13–16 ms); b-wave implicit time 28 ms (95 % reference interval: 29–33 ms); light-adapted 3.0 flicker b-wave amplitude 55/65 µV (95 % reference interval: 57–223 µV)

Full-field electroretinography (FERG) under both scotopic and photopic conditions revealed a decrease in a- and b-wave amplitudes to about 30 % of the normal values (a-waves were more distorted) as well as a- and b-wave implicit time delay. Amplitudes of oscillatory potentials were very low (Fig. 8). Electrooculogram (EOG) was normal. The Arden ratio was 2.4 and 2.2 for the RE and LE, respectively.

## Discussion

Silver deposits cause gray-blue discoloration of skin areas exposed to direct sunlight. A relationship was shown between skin pigmentation levels and the duration of exposure to silver compounds [8].

Argyrosis mainly affects people who ingest silver-based products and those who work in metal foundries, silver mines and factories with silverware [9].

Silver may accumulate in various tissues with the highest concentrations found in the skin, liver, spleen and adrenal glands [2].

At present, ocular argyrosis is less frequently diagnosed, which is associated with the withdrawal of silver-containing substances (e.g., Argyrol–silver nitrate) by drug control agencies. Nevertheless, silver-containing products are still sold as dietary supplements and ingredients in alternative medicine. This leads to the emergence of new cases of silver poisoning [10].

Confocal microscopy is a noninvasive examination facilitating the diagnosis of ocular argyrosis, especially when corneal biopsy cannot be taken. A relationship has been reported between the severity of argyrosis demonstrated on corneal confocal microscopy and advancement of generalized argyrosis [1]. Dense deposits seen in our patient hindered the visualization of the endothelium. Sánchez-Huerta et al. also encountered a problem with the assessment of this innermost layer of the cornea using confocal microscopy. Specular microscopy revealed round white bodies anterior to the corneal endothelium, whereas confocal microscopy showed bright silver precipitates with a granular pattern anterior to the corneal endothelium [11]; the precipitates resembled gold deposits [12, 13].

Spencer et al. concluded that silver deposits were inert and did not cause visual deficit [14, 15]. Some authors reported no fundus changes [13], which might have been associated with early stages of the disease.

In our patient, night blindness was related to photoreceptor damage. Fundus examination showed drusenoid changes in the macular region and RPE rearrangement, similar to the findings of Stafeeva et al. [4] and Moss et al. [8].

To our best knowledge, there are not many reports in the literature on electrophysiology testing in ocular argyrosis, and none of those we found presented detailed results of electrophysiological tests.

Stafeeva et al. described a patient with ocular argyrosis manifested with a decrease in visual acuity and nyctalopia. Electroretinography did not reveal functional changes in scotopic and photopic amplitudes, latency or in oscillatory potentials and 30-Hz flicker testing [4]. Moss et al. [8] did not observe functional deficit in scotopic, photopic electroretinography and in color vision.

On the contrary, Flögel et al. [7] described electroretinography with discretely prolonged peak times in response to 30-Hz flicker stimulation, and normal rod and cone function.

Detailed electrophysiological examinations performed in our patient did not reveal changes in RPE function; however, they revealed abnormal function of photoreceptors in the central and peripheral retina. PERG abnormalities confirmed impaired function of the inner layers of the retina in the macular region. Delayed latency of P100 wave in PVEP might have resulted from problems with visual transmission in central retina. Retinal dysfunction with abnormal ERG results might be associated with long-lasting argyrosis.

Pala mentioned numerous corneal conditions that should be taken into account in the differential diagnosis of ocular argyrosis, including pre-Descemet dystrophy, X-linked ichthyosis as well as deposits of heavy metals (iron, copper) or drugs (amiodarone, ciprofloxacin) and conditions with abnormal eye pigmentation, such as malignant melanoma [1]. If retinal changes occur, the most common alternative to ocular argyrosis is age-related macular degeneration.

## Conclusions

In the course of argyria, silver deposits may build up in various eye tissues. Confocal microscopy, OCT of the macula and electrophysiological tests are noninvasive tools, which help confirm the diagnosis of ocular argyrosis. Confocal microscopy may enable ophthalmologists to obtain in vivo cross-sectional images of the corneal layers, while OCT and electrophysiological tests may reveal retinal changes. Detailed patient history is also very important.

Until now, there have been few reports on retinal abnormalities in patients with ocular argyrosis. Changes in retinal activity occurred in photoreceptors and across to the inner layers of the retina; the most advanced changes were found in the macula. Corneal deposits and RPE changes that can cause dysfunction of the retinal adaptation to darkness and light result in considerable problems with functional vision.
